# A multi-resolution investigation for postural transition detection and quantification using a single wearable

**DOI:** 10.1016/j.gaitpost.2016.07.328

**Published:** 2016-09

**Authors:** Aodhán Hickey, Brook Galna, John C. Mathers, Lynn Rochester, Alan Godfrey

**Affiliations:** a Institute of Neuroscience, Newcastle University, Newcastle upon Tyne, UK; b Newcastle University Institute for Ageing, Newcastle University, Newcastle upon Tyne, UK; c Institute of Cellular Medicine, Newcastle University, Newcastle upon Tyne, UK; d Human Nutrition Research Centre, Newcastle University, Newcastle upon Tyne, UK

**Keywords:** Postural transition, Accelerometer, Wearables, Wavelet, Discrete wavelet transform

## Abstract

**Background:**

Multi-resolution analyses involving wavelets are commonly applied to data derived from accelerometer-based wearable technologies (wearables) to identify and quantify postural transitions (PTs). Previous studies fail to provide rationale to inform their choice of wavelet and scale approximation when utilising discrete wavelet transforms. This study examines varying combinations of those parameters to identify best practice recommendations for detecting and quantifying sit-to-stand (SiSt) and stand-to-sit (StSi) PTs.

**Methods:**

39 young and 37 older participants completed three SiSt and StSi PTs on supported and unsupported chair types while wearing a single tri-axial accelerometer-based wearable on the lower back. Transition detection and duration were calculated through peak detection within the signal vector magnitude for a range of wavelets and scale approximations. A laboratory reference measure (2D video) was used for comparative analysis.

**Results:**

Detection accuracy of wavelet and scale combinations for the transitions was excellent for both SiSt (87–97%) and StSi (82–86%) PT-types. The duration of PTs derived from the wearable showed considerable bias and poor agreement compared with the reference videos. No differences were observed between chair types and age groups respectively.

**Conclusions:**

Improved detection of PTs could be achieved through the incorporation of different wavelet and scale combinations for the assessment of specific PT types in clinical and free-living settings. An upper threshold of 5th scale approximations is advocated for improved detection of multiple PT-types. However, care should be taken estimating the duration of PTs using wearables.

## Highlights

• Evaluated wavelet algorithm for PT detection with a wearable on the lower back.
• A ‘one size fits all’ algorithm for PT is insufficient.
• Different PT types require different wavelet algorithm manipulations.
• PT detection is accurate across a number of wavelets in controlled settings.
• Temporal calculations unstable & require development prior to use in free-living.

## 1 Introduction

Physical capability tests have been shown to be predictive of all-cause mortality in older adults [1], [2]. One test is a timed sit-to-stand (SiSt) postural transition (PT) which has been identified as important for inclusion in the assessment of lifestyle-based interventions [3]. The high mechanical output needed for successful completion makes it a suitable surrogate marker of lower limb functional strength [4]. Traditionally, PTs are assessed by an observer with the use of a stop watch. Recent advances in accelerometer-based wearable technology (wearables) algorithms/methodologies have facilitated more objective measures through instrumentation in both controlled and free-living settings [5]. Wearables afford the researcher high precision temporal data as well as numerous novel accelerometer derived outcomes leading to more refined analysis [6], [7]. However, further ‘fine-tuning’ of the former has been advocated to classify and assess this activity type [8].

Detecting PT-type and duration can be achieved using sensor integration (accelerometer and air pressure) [9]. In contrast, a single sensor configuration has been utilised from a chest worn wearable using scalar product and vertical velocity estimates [10]. Alternatively, algorithms have included multi-resolution approaches where wavelets are used to detect and quantify PT performance descriptors [11], [12]. This methodology has also been applied to the signal vector magnitude (SVM) from a wearable worn on the lower back [13], proving useful when examining only PTs in composite measures of physical capability (e.g. timed-up-and-go) by supressing other signal dependant activities such as gait [14].

Wavelet-based approaches use a discrete wavelet transform (DWT), which can be interpreted as a filter bank where the signal is decomposed into several components, each representing a single frequency (scale) sub-band of the original signal [15]. Scaling of the wavelet enables frequency resolution and the shifting provides the time information [16]. This is important when considering the signal characteristics exhibited by the PT of interest, as the frequency banding must be of sufficient range to capture the desired output. As such, choice of wavelet and its associated parameters can influence the accuracy of PT detection/quantification.

Selection of wavelet methodologies (wavelet, order, and scale) often seems arbitrary with little rationale provided for the approaches taken with respect to PT analysis. To the authors knowledge no previous study has justified wavelet (and associated parameter) selection, i.e. a ‘one size fits all’ approach is usually adopted whereby a single wavelet is used for analysis of multiple PT types rather than considering different wavelets for different PTs (e.g. SiSt/StSi) [11], [12], [13]. Considering that different PT strategies are employed for different transitions [17], and the inherent effect sitting posture has on strategy selection [18], we hypothesise that a more optimal combination of wavelet and scale approximation could exist.

Therefore, the aim of this study is to examine the effect of wavelet methodology on detecting and quantifying the duration of PTs undertaken with two chair types in a large cohort of younger and older adults using a wearable on the lower back. The effect of age and chair type was also examined to investigate algorithm robustness. Manipulation of these parameters should reveal valuable information regarding best practice recommendations for analysis of PTs using wavelets. This research will serve to inform the design of wearable algorithms and direct future PT examination in clinical and free-living settings.

## 2 Methods

### 2.1 Participant recruitment

Participants were recruited from staff and students at Newcastle University and VOICENorth,^1^ an older adult volunteer group who participate in research. Participants were included only if they were healthy i.e. had no physical or neurological disabilities that might impede their movement or balance. Eighty healthy adults aged 20–40 years (40 young participants, YP) and 50–70 years (40 older participants, OP) were recruited. Ethical consent for the project was granted by the National Research Ethics Service (County Durham and Tees Valley) and the Newcastle-upon-Tyne Hospitals NHS Foundation Trust (11/NE/0383). All participants gave informed written consent before completing the study.

### 2.2 Equipment

Each participant wore a single tri-axial accelerometer-based wearable (Axivity AX3, York, UK) located on the lower back (5th lumbar vertebra-L5). The wearable was held in place by double sided tape and Hypafix (BSN Medical Limited, Hull, UK) and programmed to capture at 100 Hz (16-bit resolution, range of ±8 g). Data were stored locally on the wearable’s internal memory as a raw binary file that was downloaded upon completion of the testing session. Video recordings (Sony HandyCam DCR-SR77, Sony Europe Ltd, Surrey, England; 25 Hz) were used as a reference measure to validate the PT type (SiSt or StSi) and duration.

### 2.3 Experimental protocol

Wearable data were transformed to a horizontal-vertical coordinate system [19]. For both chair types participants completed three SiSt and three StSi transitions with short (<3s) intermittent breaks between trials as part of a scripted laboratory protocol. Prior to performance, participants were instructed to sit in a comfortable upright position. In order to preserve parity between testing measures, SiSt and StSi transitions were defined as follows for video analysis [20]:

• SiSt transition: time interval between initial vertical movement of the waist (lateral aspect of the iliac-crest) to maximal hip extension.
• StSi transition: time interval between initial downward movement of the waist to touch-down on the chair surface.

The rationale being that this definition of a PT best equated to the functionality of the wearables instrumentation, i.e. wearable location and algorithm functionality. PTs were performed from two chairs of similar height:

• Supported Chair (Chair-S): Height 0.41 m with arm-rests. Participants were instructed to use the arm-rests if they wished.
• Unsupported Chair (Chair-US): Height 0.43 m, no arm-rests.

Two-dimensional motion analysis was completed using Kinovea motion analysis software (Version 0.8.15, Kinovea, France; temporal resolution 0.04s). To avoid inter-rater error, a single researcher examined transition kinematics and derived transition durations for all individual PT performances.

### 2.4 Algorithms

After testing, data were downloaded and analysed using a bespoke Matlab^®^ program using the wavelet toolbox. A DWT was applied to the SVM of the accelerometer to extract single PTs. The DWT is given in Eq. (1) in terms of its recovery transform, where *d(k, l)* is a sampling of the wavelet coefficients at discrete points *k* and *l* with the wavelet ψ [10].

(1) $$x\left(t\right)=\sum_{k=-\infty }^{\infty }\sum_{l=-\infty }^{\infty }d\left(k,l\right)2^{-\frac{k}{2}}\psi \left(2^{-k}t-l\right)\gamma$$

The transition duration and type were estimated from twice the time and order between the negative/positive peaks, respectively [13].

### 2.5 Wavelets and scale approximation

**Fig. 1:**
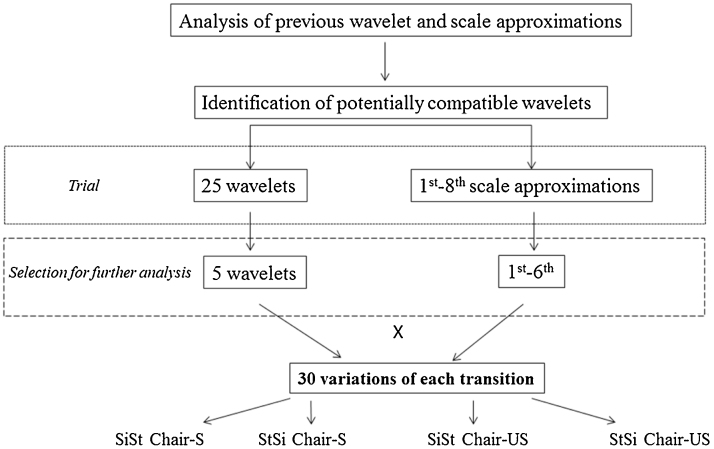
Flowchart demonstrating the process of wavelet and scale approximation selection.

A range of wavelets and orders were implemented to examine their suitability. Preliminary analysis revealed particular wavelets, orders and scales had insufficient compatibility to define the characteristics required for PT detection and duration (Fig. 1). Therefore only five wavelets and scales one-to-six were compared within this study (denoted by * Table 1.). Data were generated for the previously described PT and chair types (SiSt-S, StSi-S, SiSt-US, and StSi-US) for each participant.

### 2.6 Statistical analysis

#### 2.6.1 Detection agreement

Detection accuracy of the wavelet and scale approximation combinations compared to video were calculated for SiSt and StSi PTs. Detection accuracy of each wavelet was defined as the number of true PTs detected/(true PTs detected + PT falsely detected + PTs not detected). Kruskal-Wallis H tests and Bonferroni adjusted post hoc Mann-U Whitney tests were used to examine the differences (non-parametric) in detection accuracy of algorithm manipulations grouped by wavelet and scale approximation.

#### 2.6.2 Duration bias and absolute agreement

The magnitude of error (bias) in PT duration derived from the wearable and reference video was tested using one-sample *t*-tests. The bias was calculated as the PT duration as measured using wavelets minus that derived from the video. We took the mean difference of the three PTs per person and because of considerable skewness we expressed bias as the median of difference across the each group. Pearson’s correlations and intra-class correlations (absolute agreement on average measures, ICC_2,k_) were used to assess relative and absolute agreement between the two measures respectively. The percentage variance explained refers to the *r*^2^ from the Pearson’s correlation. We have indicated in the results (Section 3.3) where we are referring to r^2^ to avoid confusion when referring to variance explained.

#### 2.6.3 Grouped differences: age and chair

Wilcoxon matched pairs tests were used to examine the differences between chair types and independent *t*-tests were used to calculate differences between age groups.

## 3 Results

### 3.1 Demographics

Thirty-nine YP and (mean ± SD; 28.7 ± 5.4 years; 73.0 ± 14.0 kg; 1.72 ± 0.09 m) and 37 OP participants (mean ± SD; 63.9 ± 4.8 years; 71.2 ± 15.2 kg; 1.66 ± 0.09 m) completed this investigation. Four participants were removed from the analysis due to loss of wearable data.

### 3.2 PT detection accuracy

**Fig. 2:**
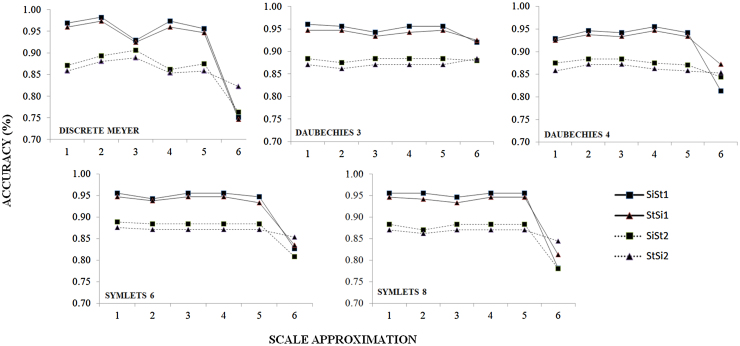
Accuracy of wavelet and frequency for detection of SiSt and StSi postural transition types (‘1’-denotes supported chair transitions, ‘2’-denotes unsupported chair transitions).

SiSt transitions elicited between 87 and 97% detection accuracy across all conditions (algorithm variation and chairs), compared with 82–86% for StSi transitions respectively. No differences were observed in the accuracy of PT detection between wavelets regardless of transition type (χ ≤ 3.554, *p* ≥ 0.492). Examination of the detection accuracy of wavelet and scale combinations (see Fig. 2) showed no differences between 1st and 5th approximations but marked differences for 6th estimations in all respective transition types. However, pairwise comparisons showed no significance (1st–5th: *Z* ≤ −0.111, *p* ≥ 0.040; 6th; Z ≤ 2.619, *p* ≥ 0.008) assuming a Bonferroni adjusted alpha level of 0.003.

### 3.3 PT duration bias and agreement

The bias differed greatly between transition types; 0.017–0.260 s for 1st–5th, and 0.080–0.408 for 6th respectively. Three transitions (SiSt-S, StSi-S, and StSi-US) displayed significant correlations for 1st-5th scale approximations (*r* *=* 0.308–0.505, *p* ≤ 0.05). The 6th order scale displayed similarly significant relationships for StSi-S (*r* = 0.281–0.425, *p* ≤ 0.05). Although significant relative agreement is observed both PTs demonstrated high variance (*r^2^* *=* 18–29% respectively).

ICC_2,k_ yielded significant results for three transition types (SiSt-S, StSi-S, and StSi-US) at 1st–5th approximations (ICC_2,k_ = 0.151–0.170, *p* ≤ 0.05). However, the poor ICC_2,k_ values suggest modest agreement between video and wearable measures. Similarly, although some combinations approached significant agreement, poor ICC_2,k_ values were also established for 6th order wavelets (ICC_2,k_ ≤ 0.063, *p* ≥ 0.056). None of the 120 permutations demonstrated acceptable agreement with the reference measure, Table 2.

### 3.4 Effect of age and condition

For SiSt transitions, no significant differences were observed in the performance of wavelet/scale combinations for Chair-S and Chair-US (*t* ≤ 0.549, *p* ≥ 0.123). Similarly, 95% of these algorithm manipulations (excluding db3 at 5th and 6th scale approximations) did not show difference according to chair type for StSi transitions (*t* ≤ 1.903, *p* ≥ 0.061). Differences in algorithm performance between age groups were only identified in the StSi transitions for Chair-US (*t* ≤ 3.295, *p* ≤ 0.021), all other conditions showed no age effect (*t* ≤ 1.914, *p* ≥ 0.60).

## 4 Discussion

This study has provided data to inform appropriate choice of wavelet and scale for successful PT detection and quantification from a single wearable on L5. It is the first study to systematically compare a comprehensive range of wavelet and scale combinations to detect PTs, and to assess these combinations across chair types in a large group of young and older adults. Although detection of PTs shows promise, estimating the duration of PTs is subject to bias and lacks both relative and absolute agreement with the reference measure. These findings will help inform development of wearables worn on L5 to improve the classification of postures and contextualisation of general activities of daily living.

### 4.1 PT detection

These results indicate that PTs can be successfully detected using a single wearable on L5 as a component of a scripted laboratory protocol, a premise consistent with previous research [20], [21]. Accuracies of >82% were achieved regardless of the wavelet chosen, but algorithm performance was increased up to 97% for SiSt when using 1st–5th scale approximations. Reduced accuracy was observed for StSi transitions which may be attributed to the greater variety of kinematic strategies employed by participants in this body mass lowering manoeuvre [17], however this requires further investigation [22].

### 4.2 PT duration

Previous findings [20] examining young and older adults reported acceptable mean biases of 0.01–0.16 s between accelerometers and reference measures. Both PT types in this study demonstrated comparable median differences; SiSt (1st–5th) 0.01–0.05 and StSi (6th) 0.08–0.17 s respectively. However our results indicate that estimates of PT duration show poor relative and absolute agreement (Table 2). Although comparable, the lack of statistically significant agreement may result from the heterogeneity of our larger sample sizes, and as such may be a more accurate representation of accelerometer function. This study utilised a single rater and clearly defined PT to minimise error in PT duration. Thus, methods for potentially improving calculations still require further consideration, such as 3-dimensional motion analysis.

### 4.3 Age and condition

We found no difference in PT duration between supported and unsupported chair types and in addition, there was little evidence of differences between age groups with only one transition condition displaying noteworthy difference between young and older participants. It would appear that despite the potential effects of transition strategy or age-related physical function a level of robustness for all algorithm combinations has been observed. However, this warrants further investigation in relation to identifying PTs in uncontrolled environments.

### 4.4 Wavelet and scale approximation selection

The lack of statistical difference between wavelet types provides interesting insight into the selection criteria previous stated in section 2.5. It appears that wavelet selection could be considered negligible. Of greater interest is the differences observed in scaling parameters (i.e. frequencies). Understanding the differences in sensor location and performance should help explain these differences. For example, Najafi et al. [12] used a chest worn monitor (40 Hz), and employed a Coiflet wavelet of order five at 5th-9th scales, corresponding to a frequency band of 0.04-0.068 Hz. In this study, a 100 Hz wearable coupled with the lower amplitude of the acceleration signals captured from L5 required a larger frequency band. As such, the need to consider sensor location/function, and the frequency output of the movement to be analysed is crucial for accurate PT analysis. This helps clarify the incompatible nature of any signal decomposed at 6th approximations or greater (Fig. 2), and why pelvic/lumbar wearable locations appear to have a defined threshold of 5th scale approximations.

With respect to algorithm functionality, PT detection, 1st–5th scale approximations demonstrated proportionally lower error (Table 1). Therefore, our results support previous research which advocates use of a single set of wavelet and scale parameters [11], [12], [13]. Although suitability has been confirmed for detection, we did not find a single set of parameters that was adequate for estimating durations. A combination of algorithmic methods, i.e. the implementation of ‘rising/SiSt’ detectors and ‘lowering/StSi’ detectors could be more suitable to provide approximations of temporal characteristics. This could be of particular importance for detecting and quantifying PTs in free-living settings and should be a focus of future investigations.

### 4.5 Strengths and limitations

This paper is the first to provide data to support the choice of wavelet and scale for PT analysis via accelerometry. The large variation in algorithm inputs for different chair types and age groups is a strength of this investigation.

It is possible that limitations in the study design reduced our ability to detect significant agreement between wavelet-based calculations and the reference method. This study has sought to examine single/intermittent PTs whereas previous investigations have estimated temporal characteristics of repeated transition functional mobility tests, namely; three and five-times-sit-stand. The calculation of temporal characteristics across multiple transition examinations will reduce bias relative to the number of observed transitions. For example, Godfrey et al. [14] calculated PT duration (stopwatch v. wearable) from initial trough to the final peak of a series of transitions with a correction factor of 1.4. Whereas this study (video v. wearable) more directly examines individual transitions, calculating the PT duration as twice the difference between negative and positive peaks, eliminating corrections for multiple PTs. As such, the pursuit of individual PT analysis in this study may serve to amplify the relative effects of error and reduce significance.

**Fig. 3:**
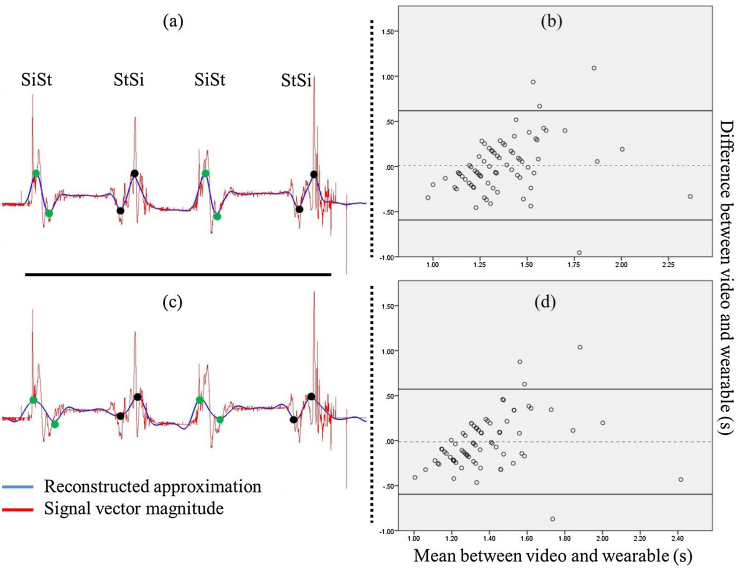
(a) Example of a series of PTs for a participant with SVM (red trace) and reconstructed approximation (blue) from a 3rd scale 4th order Daubechies wavelet and peaks of interest, (b) its corresponding Bland-Altman plot for all participants. (c) Single participant PTs SVM (red) and approximation (blue) from a 6th scale 5th order Symlet wavelet, (d) its corresponding Bland-Altman plot for all participants. In the Bland-Altman plots the horizontal dotted line indicates no difference between PT duration assessed using the video and wearable. Solid lines indicate the 95% limits of agreement for the difference. (For interpretation of the references to colour in this figure legend, the reader is referred to the web version of this article.).

The specific kinematic identifiers of SiSt and StSi ‘start’ and ‘end’ points that have been applied for PT detection may in fact be too constrained for quantification of temporal parameters, and do not satisfy the range of transition strategies employed by participants. This may suggest why four outliers were observed, Fig. 3. Future investigations could revise these PT descriptions and may consider adopting different signal characteristics for algorithm implementation. The different sampling frequencies employed by the accelerometer and reference measure limit the compatibility of resultant temporal information, and the assumption of photogrammetry as a ‘gold-standard’ may require re-evaluation and could be replaced with 3-dimensional positional analysis. However, observing the successful accuracy results and the minimal absolute errors observed between measures, these limitations are considered as appropriate, but should be noted in future assessments.

## 5 Conclusion

We have shown that PT detection via accelerometry/DWT is accurate across a number of wavelets in controlled settings. A newly defined upper threshold of 5th scale approximation has been established for more accurate detection of multiple PTs from L5. These methods proved unreliable for estimation of PT duration. Participant age and chair type had minimal influence on algorithm performance. Due to the inherent differences in kinematic strategies of SiSt and StSi respectively, it is recommended that studies extracting PT durations from acceleration signals at L5 should explore the use of different wavelet parameters for different PT types. Future research should investigate the use of these recommendations for PT detection and quantification in uncontrolled environments and in pathological groups.

## Conflict of interests

None.

## Author contributions

All authors contributed to the design and implementation of the study. AG was responsible for data collection and Matlab^®^ scripts. AH completed all processing of acceleration and 2-dimensional video data. AH, BG, AG all contributed to data analysis and interpretation. JCM and LR provided comment on the manuscript. All authors contributed to the revision of the manuscript and approved the final version for publication.

## Funding

JCM led, LR was an investigator and AG supported the LiveWell program a research project funded through a collaborative grant from the Lifelong Health and Wellbeing (LLHW) initiative, managed by the Medical Research Council (MRC) on behalf of the funders (grant number: G0900686). LR and AG are supported by the National Institute for Health Research (NIHR) Newcastle Biomedical Research Centre (BRC) and Unit (BRU) based at Newcastle upon Tyne Hospitals NHS Foundation Trust and Newcastle University. The views expressed are solely those of the authors.

## Figures and Tables

**Table 1:** **DWTWavelets**, wavelets implemented into the Matlab^®^ based DWT for detection and quantification of PTs.

Family of wavelets	Order
Daubechies	db1, db2, db3*, db4*, db5, db6
Coiflets	coif1, coif2, coif3, coif4
Symlets	sym1, sym2, sym3, sym4, sym5, sym6*, sym7, sym8*
Mexican Hat	mexh
Mayer & Discrete Meyer	meyr, dmey*
Gaussian	gaus1, gaus2, gaus3, gaus4

* Denotes the compatible wavelets that were taken for further analysis.

**Table 2:** **Wavelet Performance,** descriptive, accuracy, bias and agreement, and interaction results for 1st-5th and 6th scale approximations respectively.

Transition type	Scale approximation	Difference	Detection accuracy	One sample *t*-test		Correlation/Agreement					Interactions	
		(average of medians)	(mean%)	T	p	r	p	ICC	LoA		Z	p
									*min*	*max*		
SiSt chair 1	1st–5th	−0.051	95	≤0.385	≥0.261	≤0.466	≤0.002*	≤0.154	−0.653	0.640	≥−2.348	≥0.040
	6th	−0.407	82	≤−7.445	≤0.0005	≤0.184	≥0.114	≤0.020	−1.639	0.640	≤−2.619	≥0.008
StSi chair 1	1st–5th	0.260	94	≤5.901	≤0.0005	≤0.505	≤0.0005*	≤0.170	−0.619	1.174	≥−2.184	≥0.040
	6th	−0.080	84	≤0.779	≥0.129	≤0.425	≤0.014	≤0.063	−0.970	1.026	≤−2.546	≥0.008
SiSt chair 2	1st–5th	0.017	88	≤1.197	≥0.235	≤0.212	≥0.068	≤0.053	−0.672	0.738	≥−1.342	≥0.556
	6th	−0.408	82	≤−7.710	≤0.0005	≤0.216	≥0.062	≤0.026	−1.202	0.383	≤−2.117	≥0.040
StSi chair 2	1st–5th	0.177	87	≤5.026	≤0.0005	≤0.368	≤0.007*	≤0.088	−0.611	1.028	≥−1.678	≥0.278
	6th	−0.083	86	≤−0.597	≥0.160	≤0.204	≥0.079	≤0.026	−0.955	0.810	≤−1.392	≥0.040

## Footnotes

1 www.ncl.ac.uk/changingage/engagement/VOICENorth
